# Unilateral cross-incompatibility between *Camellia oleifera* and *C. yuhsienensis* provides new insights for hybridization in *Camellia* spp

**DOI:** 10.3389/fpls.2023.1182745

**Published:** 2023-07-03

**Authors:** Han Gong, Yihong Chang, Jinming Xu, Xinran Yu, Wenfang Gong

**Affiliations:** Key Laboratory of Cultivation and Protection for Non-Wood Forest Trees of Ministry of Education, Central South University of Forestry and Technology, Changsha, China

**Keywords:** interspecific hybridization, pistil-pollen interaction, pre-zygotic barrier, semi *in-vivo test*, calcium

## Abstract

*Camellia yuhsienensis* was used to cross with *Camellia oleifera* to improve the resistance of oil camellia anthracnose. However, unilateral cross-incompatibility (UCI) between *C. oleifera* and *C. yuhsienensis* was found during the breeding process. Five *C.oleifera* cultivars and four *C. uhsienensis* materials were tested to confirm the UCI between *C. oleifera* and *C. yuhsienensis*. ‘Huashuo’ (HS) and ‘Youza 2’ (YZ2) were used to represent these two species to characterize the UCI, including pollen tube growth, fertilization and fruit development. The results demonstrated that UCI was prevalent between *C. oleifera* and *C. yuhsienensis*. The asynchronous flowering period was a pre-pollination barrier that limited mating between these two species under natural conditions. Interspecific pollen tubes were observed through the styles of these two plants, though the growth rates differed considerably. At 96 hours after pollination, the pollen tube of YZ2 barely entered the ovule, but remained at the base of the style and became swollen. However, the HS pollen tube entered the ovule 48 hours after pollination, double fertilization was observed, and the fruit and seeds developed commonly. Relative to compatible combinations, most unfertilized ovules in incompatible combinations failed to grow, turned brown 150 days after pollination, and the fruits were smaller than expected with uneven enlargement. Investigations on both semi-*in vivo* and *in vitro* pollen tubes gave us new idea for thought: the HS style has a stronger inhibitory effect on the interspecific pollen tubes, while calcium alleviates the inhibitory of styles but failed to prevent the appearance of abnormal pollen tube morphology. This study provides useful information on interspecific hybridization between *C. oleifera* and *C. yuhsienensis* for understanding reproductive isolation mechanisms and breeding programs in genus *Camellia*.

## 1 Introduction

*Camellia oleifera* Abel, belongs to Sect. *oleifera* of the genus *Camellia*, whose seeds can produce oil with economic benefit, is one of the dominant woody oil crops widely cultivated in southern China. *C. oleifera* has large fruits with rich oil content but is sensitive to anthracnose (Wang et al., 2017; Li et al., 2021; Quan et al., 2022). *Camellia yuhsienensis* Hu, a relative of *C. oleifera* in the genus *Camellia*, is characterized by high oil quality and strong resistance to anthracnose, a source of natural resistance for use in elite cultivars (Chen et al., 2021; Li et al., 2021). Crossbreeding is a crucial part of the *C. oleifera* tree breeding program because of its self-incompatibility (Liao et al., 2014; Goulet et al., 2017; Lenzi et al., 2021).

Interspecific hybridization enables the introduction of new valuable traits and is an effective means of breeding new varieties, but is limited by reproductive specificity. Cross incompatibility is a mechanism of reproductive isolation categorized as unilateral or bilateral (Maune et al., 2018). Unilateral cross-incompatibility(UCI) is defined as asymmetric reproductive isolation, manifested by successful crosses in one direction and unsuccessful crosses in the other (Muñoz-Sanz et al., 2020). Currently, UCI has been observed in banana shrub (Xu et al., 2017), jacaranda tree (Bittencourt, 2019), azalea (Okamoto and Ureshino, 2015), etc. However, no studies have been reported on the cytological mechanism of interspecific asymmetric hybridization in the genus *Camellia* between *Camellia oleifera* and *Camellia yuhsienensis.*

Hybrid incompatibility is generally classified into pre- and post-pollination barriers (Moyle et al., 2014). Pre-pollination barriers consist of differences in flowering period, flower morphology, and pollinators formed by the interaction between the environment and the species (Muñoz-Sanz et al., 2020). The post-pollination barriers are divided into pre- and post-zygotic barriers based on whether they are fertilized or not, which triggers the accumulation of genetic differences during the process of speciation (Baack et al., 2015). Both post-pollination barriers reduce the fruit set, even though the potential mechanisms are distinct (Lowry et al., 2008). Pre-zygotic barriers after pollination are related to pollen-pistil interactions, including the inability of pollen to germinate on the stigma and the inability of the pollen tube to reach the embryo sac to complete fertilization successfully (Moyle et al., 2014). Even after completing double fertilization, plants face post-zygotic barriers such as embryo abortion, embryo developmental failure, and offspring sterility (Chen et al., 2016).

Calcium is essential for the germination and growth of pollen tubes, whose role during pollination and fertilization has received particular attention (Zheng et al., 2019). Previous studies reported that a specific calcium distribution existed at the tip of the fast-growing pollen tube (Miller et al., 1992). Moreover, the semi-*in vivo* technique has recently been widely used to study pollen-pistil interactions (Hafidh et al., 2014; Claessen et al., 2022). However, it has not been applied to interspecific cross-compatibility within the genus *Camellia*.

In this study, UCI was found in *C. oleifera* and *C. yuhsienensis*. *C. oleifera* cultivar ‘Huashuo’ (HS) and *C. yuhsienensis* cultivar ‘Youza 2’ (YZ2) were used as representatives to identify the cytological mechanisms of UCI. Besides, the exploration of interspecific semi-*in vivo* system clarified that the inhibition of interspecific pollen tube was mainly due to the style. Based on the results, we discussed the possibility of improving *C. oleifera* cultivars by interspecific hybridization, which provides a reference for overcoming the incompatibility of genus *Camellia* crosses.

## 2 Materials and methods

### 2.1 Plant materials

Five *C. oleifera* cultivars ‘Huajin’(HJ), ‘Huaxin’(HX), ‘Huashuo’ (HS), ‘Xianglin’ (XLC15), ‘Changlin18’(CL18) were selected. Similarly, a cultivar ‘Youza 2’(YZ2), and three superior individual plants YZ1, W1, and W2 were selected among *C. yuhsienensis*. The above nine plant materials were used for interspecific hybridization. *C. oleifera* cultivars are five-year-old grafted seedlings and *C. yuhsienensis* are ten-year-old natural trees, which were mature trees with stable characteristics. The plant material was planted and grown at the Central South University of Forestry Science and Technology (lat. 28°05′N, long. 113°21′E), in a humid subtropical monsoon climate zone with an average annual temperature of 18.2°C and annual precipitation of 1715.8 mm.

### 2.2 Characteristics of pre-pollination barriers

Single plants of five cultivars of *C. oleifera* were randomly selected for flowering investigation with four individual plant materials of *C. yuhsienensis* to investigate the flowering period. The flowering periods of *C. oleifera* and *C. yuhsienensis* in 2021-2022 were recorded, and the flowering status was described by three periods: the beginning of flowering, full flowering, and end of flowering, indicating that the flowering amount reached 5%, 25%, and 95% of the plant (Wei et al., 2021).

### 2.3 Characteristics of post-pollination barriers

#### 2.3.1 Artificial pollination

Artificial pollination included interspecific crosses between *C. oleifera* and *C. yuhsienensis*. Two interspecific hybrid combinations “*C. oleifera* × *C. yuhsienensis*” and “*C. yuhsienensis* × *C. oleifera*” were set up. A fixed pollen parent was employed in each combination and pollinated separately with a different variety of the other species, with each combination pollinating 50 flowers. Flowers were selected at the bud stage, emasculated before pollination, then the viable pollen was manually applied to the stigma. After completing pollination, the flowers were covered with sulfate paper bags until 7 days after pollination (DAP). The fruit set rate was counted every 30 d within 270 DAP, dividing the number of expanded ovaries by the number of pollinated flowers. And the cross-compatibility index was calculated by dividing the seed number by the number of pollinated flowers.

#### 2.3.2 Pollen tube observation

*In situ*: Two cross-pollinated combinations, “HS × YZ2” and “YZ2 × HS”, were used as representatives for the histological observation. Pistils were collected after 3, 6, 12, 18, 24, 36, 48, 72, 96, 120, 144, and 168 hours after pollinations (HAP), treated with NaClO solution (active chlorine 9000 mg·L^-1^) for 0.5 h, softened with 8 mol·L^-1^ NaOH for 1 h, and stained with 0.3% (w/v) aniline blue and observed by fluorescence microscopy (Olympus BX51, Japan). Observation on pistils was at least 5 in each period, and the number of pollen tubes *in situ* observed was n ≥ 45.

*In vitro*: Collected pollen from HS and YZ2 was removed from -80°C and placed at normal temperature (25°C) for 1 h. Pollen was incubated on solid medium, consisted of 10% sucrose, 0.01% boric acid, 1% agar (w/v).Pollen tube lengths were counted and recorded at 0-12 h every 2 h and at 12-48 h every 4 h. The number of pollen tubes *in vitro* observed was n ≥ 20.

#### 2.3.3 Embryology observation

Ovules were stained with eosin for 1 h, dehydrated by ethanol gradient, and transparent with methyl salicylate (Gao et al., 2018). Confocal laser scanning microscope(CLSM) obtained images with an excitation wavelength of 512 nm (Leica SP8, Germany), combined with conventional observation of 9 μm paraffin sections, for investigating the double fertilization of each ovule.

### 2.4 Pollen tube growth inhibition mechanism

#### 2.4.1 Semi-*in vivo* trial

Semi-*in vivo* culture conditions were explored containing style and ovary factors. Styles of different lengths were cut and divided into three groups: top, middle, and bottom, indicating the top third of the style, half of the style, and the entire style separately. The presence or absence of ovary below the style incision were the two treatments explored for the ovary factor.

Pistils were retrieved immediately after pollination and treated as previously reported (Chang et al., 2023). The treated styles and ovaries were placed on a solid medium for culture. The medium composition was the same as pollen culture *in vitro* with the addition of an additional 50 mg·L^-1^ CaCl_2_. The film separated the pollinated stigma from the medium, and the style incision was placed tightly against the medium. After incubation in the dark at 25°C, the style was observed microscopically. The length and number of pollen tubes growing out of the style incision were observed and recorded.

#### 2.4.2 Ca^2+^ fluorescent probe loading

[Ca^2+^]_cyt_ of pollen tubes grown *in vitro* (HX and YZ2 pollen cultured in 10% sucrose, 0.01% boric acid) and semi-*in vivo* were labeled with Fluo-4/AM ester at -80 kPa, 4°C, in the dark for 30 min (Li et al., 2018), and then washed three times with basic culture solution. After that, the labeled pollen tubes were left at room temperature for 30 min and observed at 488 nm excitation wavelength under the CLSM.

### 2.5 Statistical analysis

Statistical scores were recorded and analyzed using Microsoft Excel 2019 and GraphPad Prism 8.0. Significance analysis of differences between two samples was assessed using a Student’s *t*-test or Duncan’s test, and *p* < 0.05 was considered statistically significant.

## 3 Results

### 3.1 Comparison of flowering periods and the fruit set of interspecific hybridization

**Figure 1:**
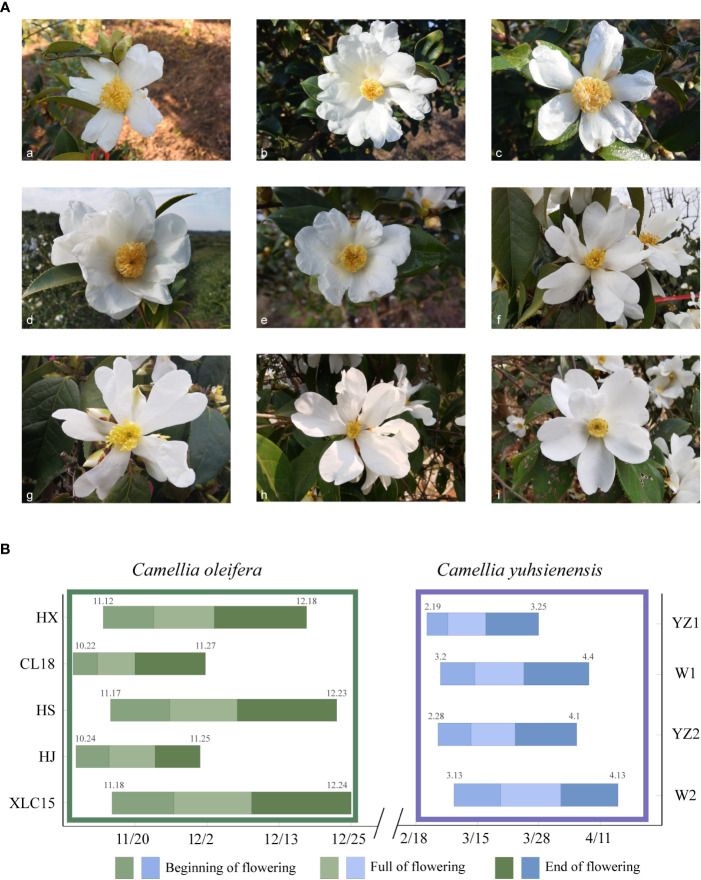
Blooming flower morphology and flowering periods. **(A)** Flowering morphology of five *C oleifera* cultivars and four *C yuhsienensis* plants used as cross-pollinated parents. (a) ‘Huajin’ (HJ). (b) ‘Huaxin’(HX). (c) ‘Huashuo’(HS). (d) ‘Xianglin’ (XLC15). (e) ‘Changlin18’(CL18). (f) YZ1. (g) YZ2. (h) W1. (i) W2. **(B)** Flowering period of *C oleifera* and *C yuhsienensis.* Among them, (a–e) are of *C. oleifera*, and (f–i) are of *C. yuhsienensis*.

The five *C. oleifera* cultivars bloomed from October to December (**Figure 1A**-(a-e)), and the four *C. yuhsienensis* cultivars bloomed from February to April (**Figure 1A**-(f-i)). Plants from these two species differed in the distribution of the beginning, full, and end of flowering stages but not in the total length of the flowering period. The total flowering period of *C. oleifera* was significantly extended than that of *C. yuhsienensis* on average 35 d and 32.5 d, respectively (**Figure 1B**).

**Figure 2:**
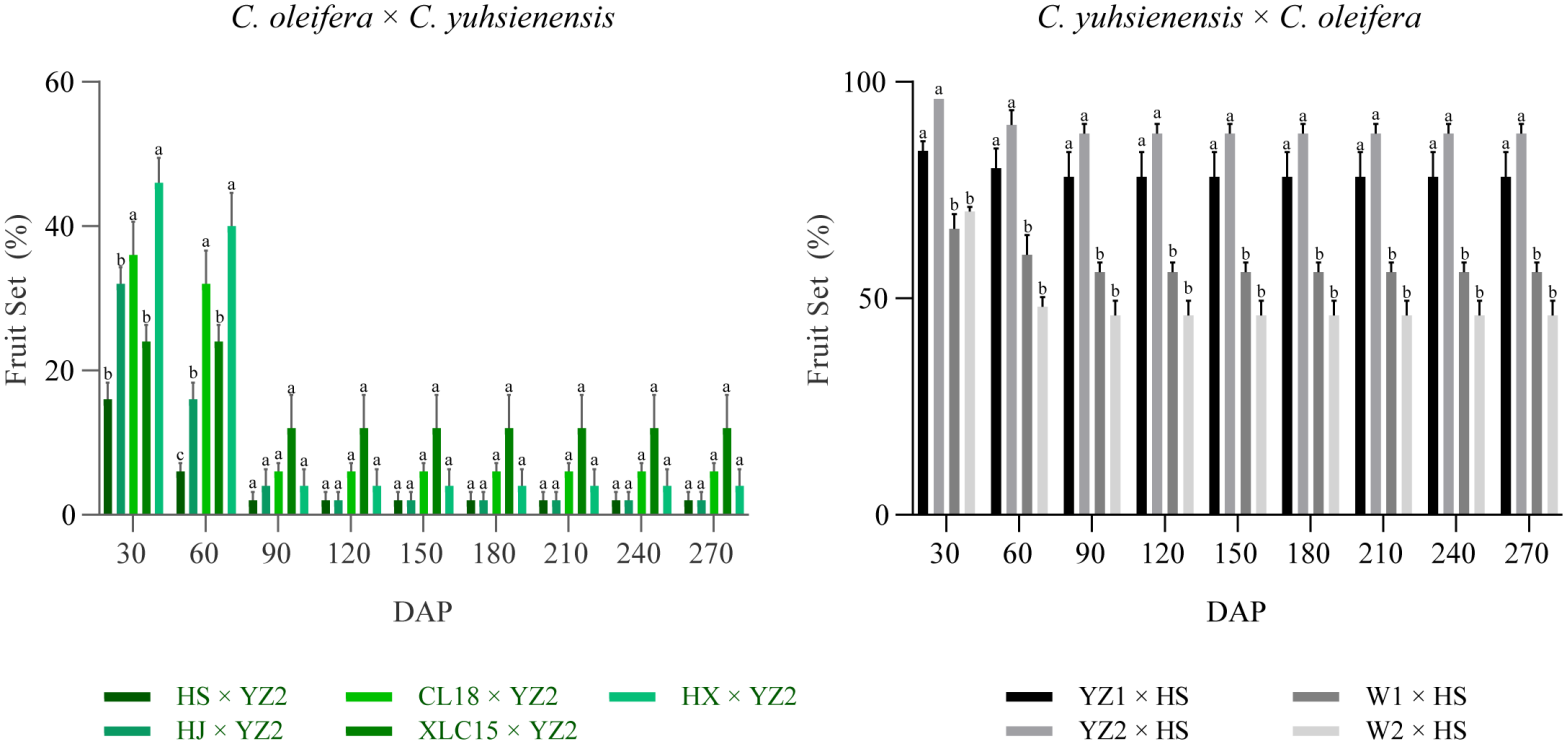
Investigation of fruit set rate in interspecific crosses between *C. olefera* and *C. yuhsienensis*. Error bars show ± SD. Different lowercase letters indicate significant differences by Duncan’s test (*p*<0.05).

Based on the survey of fruit set rate every 30 days, the fruit set rate of these two cross combinations was found to remain stable after 90 DAP (**Figure 2**). Few fruits were obtained in the hybrid combinations with *C. oleifera* as the pollen recipient, with fruit set rates of 4.0%, 4.0%, 2.0%, 8.0%, and 6.0%, respectively. While in the hybrid combination with *C. yuhsienensis* as the pollen recipient, the compatibility was better by 56.0%, 46.0%, 78.0%, and 88.0% fruit set rates, respectively. Besides, the cross-compatibility index also showed better compatibility of *C. yuhsienensis* as the recipient parent rather than the pollen donor (**Table 1**).

**Table 1:** The fruit set rate and seed production in different cross combinations between *C. oleifera* and *C. yuhsienensis*.

Female	Male	Seed number	Fruit set rate (%)	Cross-compatibility index
*C. oleifera* × *C. yuhsienensis*				
HJ	YZ2	2	4.0 ± 0.02c	0.04
HX	YZ2	2	4.0 ± 0.04c	0.04
HS	YZ2	1	2.0 ± 0.02c	0.02
XLC15	YZ2	4	8.0 ± 0.08c	0.08
CL18	YZ2	3	6.0 ± 0.02c	0.06
*C. yuhsienensis* × *C. oleifera*				
HS	W1	198	56.0 ± 0.04b	3.96
HS	W2	124	46.0 ± 0.06b	2.48
HS	YZ1	228	78.0 ± 0.10a	4.56
HS	YZ2	254	88.0 ± 0.04a	5.08

The cross-compatibility index was calculated by dividing the total number of seeds by the total number of pollinated flowers. The data on fruit set rate are means ( ± standard deviation). Different lowercase letters indicate significant differences by Duncan’s test (*p*<0.05).

### 3.2 Characterization of pollen tube growth and ovule penetration

**Figure 3:**
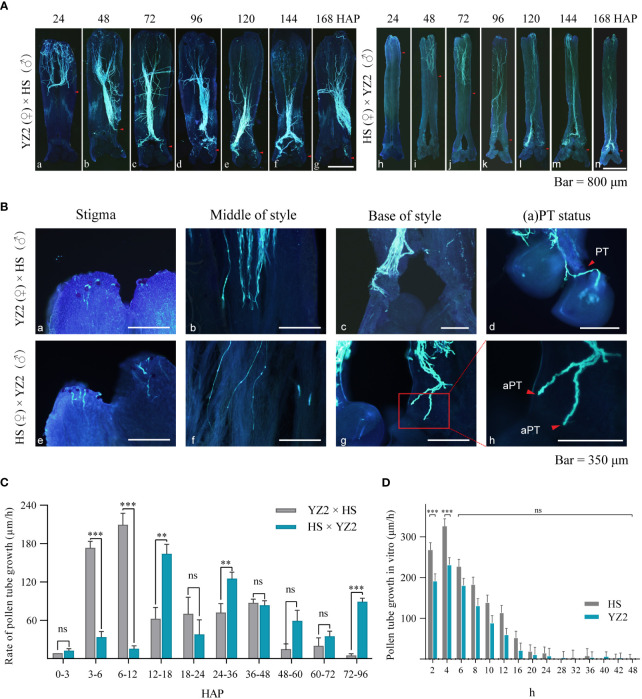
Pollen tube growth in the interspecific reciprocal cross of HS and YZ2. **(A)** Pollen tube growth pattern in interspecific crosses at 24, 48, 72, 96, 120, 144, and 168 hours after pollination (HAP). The red arrows indicate the maximum length of pollen tube growth in each period. **(B)** Dynamic growth of pollen tubes. (a–c) The pollen tube of HS on the stigma of YZ2, in the style, and growing at the base of the style. (d) The pollen tube of HS penetrated the ovule via the micropyle. (e–g) The pollen tube of YZ2 on the stigma of HS, in the style, and growing at the base of the style. **(H)** The enlarged view of the red box in (g) demonstrates the abnormal pollen tube morphology. **(C)** Comparison of growth rates at different periods before pollen tubes reached the base. Pollen tubes *in situ* were observed and counted in each period n=45. Error bars show ± SD. Significant differences at *p<0.05, **<0.01, and ***<0.001. “ns” indicates no significant difference. **(D)** Measurement of pollen tube length in HS and YZ2 grown *in vitro*. The growth rates of these species had similar trends. Pollen tubes *in situ* were observed and counted in each period n=30. Error bars show ± SD. Significant differences at ***p <0.001. “ns” indicates no significant difference. aPT, abnormal pollen tube; PT, pollen tube.

The growth patterns of pollen tubes of these two hybrid combinations were compared, with “HS × YZ2” and “YZ2 × HS” as representatives. Within the selected period, pollen tubes were observed in all parts of the style of both species (**Figure 3A**). At 24 HAP, the pollen tubes of HS grew to about one-half of the style of YZ2 (**Figure 3A**-a), while the pollen tubes of YZ2 only grew to one-fifth of the style of HS (**Figure 3A**-h). The pollen tubes in YZ2 reached the base of the style at 48 HAP (**Figure 3A**-b), while the pollen tubes in the HS style only grew to a quarter of the whole style (**Figure 3A**-i). The pollen tubes of HS grew to half of the YZ2 style at 72 HAP and reached the base of the style at 96 HAP (**Figure 3A**-j-k).

Pollens from HS and YZ2 were able to germinate on the interspecific stigma (**Figure 3B**-(a-e)) and grew to the base of the style, although at different times. Remarkably, when the pollen tubes grew to the base of the style, the pollen tubes of the HS continued to grow down to the ovary in the YZ2 style (**Figure 3B**-c). In contrast, the pollen tubes of the YZ2 stagnated at the base of the style in HS (**Figure 3B**-g), and the tip of pollen tube became swollen (**Figure 3B**-h). At the time after the pollen tube reached the base of the style (“HS × YZ2” after 96 HAP and “YZ2 × HS” after 48 HAP), the ovules of all pistils were treated transparently to observe whether the pollen tube penetrated. In the bottom style of YZ2, pollen tubes of HS grew along the hollow placenta toward the ovule and penetrated the ovule through the micropyle (**Figure 3B**-d). However, most pollen tubes of YZ2 reach only the base of the HS style (**Figure 3A**-(k-n)), with almost no pollen tubes entering the ovule, although the fruit set rate suggested this possibility.

The velocity variation of the pollen tube before reaching the base of the style was refined (**Figure 3B**-(b-f)) and the growth rate of the pollen tube *in situ* was compared between the two cross combinations (**Figure 3C**). At 3 HAP, the HS pollen tubes were already fast-growing. In comparison, the speed of YZ2 pollen tubes rose rapidly after 12 HAP, but they both grew in a conventional pattern in style. The velocity of the HS pollen tube diminished at 48 HAP due to reaching the base of the style, while the YZ2 pollen tube did not reach the base of the style, but the velocity started to decrease. Compared to the HS pollen tubes produced in YZ2, the high-speed growth period for the ones grown in HS was delayed. However, HS and YZ2 pollen tubes culture *in vitro* showed the same trend of increasing velocity until 24 h (**Figure 3D**), suggesting that the growth of YZ2 pollen tubes grown in HS styles may be restricted.

### 3.3 Observation of double fertilization and fruit development of interspecific hybridization by mass pollination

**Figure 4:**
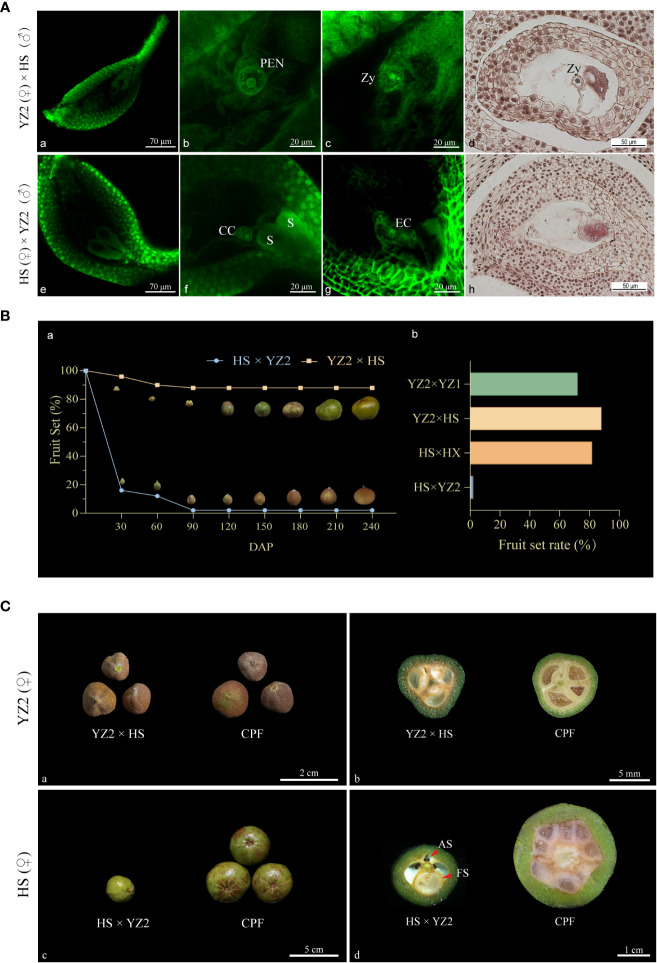
Fertilization and fruit development in the interspecific reciprocal cross of HS and YZ2. **(A)** Observations on the embryo sacs of two hybrid combinations. (a) The embryo sac of a single ovule of YZ2 was shown under the fluorescence field. (b) After 48 HAP, the primary endosperm nucleus with conidia was formed in the embryo sac of YZ2. (c) A sperm entering the egg cell in the embryo sac of YZ2 was undergoing fusion to form a zygote. (d) Paraffin sections of ovule from YZ2 showed zygote after sperm-egg fusion, observed at 60-72 HAP. (e) The embryo sac of a single ovule of HS was shown under the fluorescence field. (f) After 96 HAP, the two synergids were morphologically normal in the HS embryo sac, and the central cell with two polar nuclei was above it. (g) An unfertilized egg cell in the HS embryo sac. (h) Paraffin sections of unfertilized ovule from HS observed at 120-168 HAP. **(B)** Comparison of fruit-setting rates between intraspecific and interspecific pollination combinations. (a) Fruit set and morphological development of the two interspecific combinations ranged from 30-240 DAP. (b) Comparison of the final fruit set rate of interspecific and intraspecific pollinated combinations. “YZ2 × YZ1” and “HS × HX” are intraspecific compatible pollination combinations with *C. yuhsienensis* and *C. oleifera* as the female parent, respectively. **(C)** Comparison of fruit morphological traits of two hybrid combinations. (a) Comparison of YZ2 × HS and YZ2 compatible-pollinated fruits at 150 DAP. (b) Cross-sectional comparison of YZ2 × HS and YZ2 compatible-pollinated fruits at 180 DAP. (c) Comparison of HS × YZ2 and HS compatible-pollinated fruits at 150 DAP. Comparison of normal and abnormal seeds inside the fruit at 150 DAP. (d) Cross-sectional comparison of HS × YZ2 and HS compatible-pollinated fruits compatible pollinated fruits at 180 DAP. Red arrows indicate abnormal and fertile seeds. PEN, primary endosperm nuclei; Zy: zygote; S, synergid; CC, central cell; EC, egg cell; CPF, compatible-pollination fruit; AS, aborted seed; FS, fertile seed.

Mass pollination was performed to observe double fertilization and to obtain fruits of incompatible combinations. Ovule fertilization observations after pollination with HS and YZ2 were made when the pollen tube reached the style base. The ovules of both recipient pistils developed usually, and the embryo sac structure was intact (**Figure 4A**-(a-e)). At 48 HAP, the pollen tube of HS entered the ovule of YZ2. One of the released sperm fused firstly with the two polar nuclei to form the primary endosperm nucleus (**Figure 4A**-b), and the other sperm subsequently fused with the egg cell to form the zygote (**Figure 4A**-(c-d)). No characteristic fertilization was observed in the investigated ovules of 168 HAP from five pistils. Unfertilized ovules were found to have a complete embryo sac structure, including the egg apparatus and the central cell (**Figure 4A**-(f–h)).

Fruit development of the two crosses was followed. Even with heavy pollination, “HS × YZ2” produced few fruits, and the few remaining fruits enlarged unevenly (**Figure 4B**-a). To further characterize the interspecific fruit, fruit from compatible intraspecific pollination “YZ2 × YZ1” and “HS × HX” were introduced for comparison (**Figure 4B**-b). “YZ2 × HS” harvested many available fruits that were essentially indistinguishable in appearance from those obtained from its intraspecific compatible pollination. The remaining fruits of “HS × YZ2” had significantly smaller fruits than intraspecific hybridization with HS as a receptor (**Figure 4C**(-a-c)). At 150 DAP, “YZ2 × HS” fruit seeds were creamy white and usually developed, indistinguishable from intraspecific hybrid fruits (**Figure 4C**-b). Correspondingly, of the few left “HS × YZ2” fruits, only one ovule was fertilized and developed, slightly larger than any fertilized ovule from HS intraspecific pollination. All the rest of the “HS × YZ2” unfertilized ovules turned brown with no increase in size (**Figure 4C**-d).

### 3.4 Analysis of the causes of incompatible combination

#### 3.4.1 Exploration of interspecific semi-*in vivo* pollination experimental system

**Figure 5:**
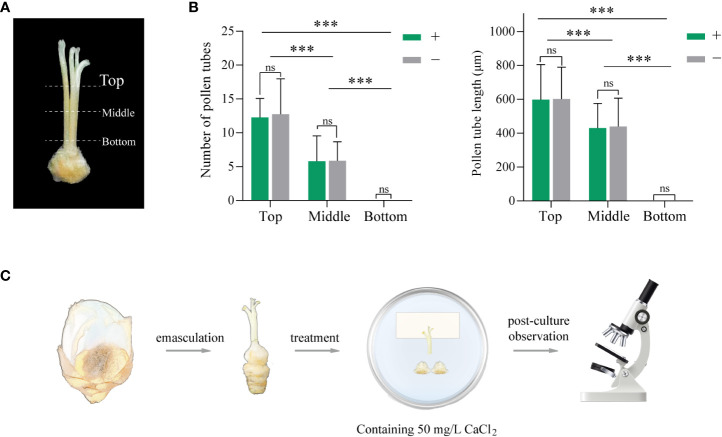
Exploration of semi-*in vivo* pollination assay of “HS × YZ2”. **(A)** Illustration of three style lengths used in the experiments. Top: the upper third of the style; Middle: half of the style, Bottom: complete style. **(B)** The number and length of pollen tubes growing out of each group of incisions. “+” and “−” represent the presence and absence of ovaries below the incision, respectively. At least n=30 styles were examined in each group. Error bars show ± SD. Significant differences at ***p <0.001. “ns” indicates no significant difference. **(C)** Diagram of the major steps of the semi-*in vivo* pollination test. The medium supplemented with 50 mg·L^-1^ CaCl_2_ alleviated the inhibition of the style and allowed the pollen tubes to grow out at the incision of the intact style.

An attempt *in vivo* was made to analyze the reason why the pollen tubes of YZ2 were inhibited by the HS pistil. To simulate the pollen tube growth *in situ* of “HS × YZ2”, an exploration of semi-*in vivo* culture was conducted. Semi-*in vivo* culture conditions were explored containing style and ovary factors. Style factors were explored by examining the length and number of pollen tubes grown in three different lengths of style incisions (**Figure 5A**). After 12 HAP, pollen tubes emerged from the incision of the “Top” style, while after 24 HAP, pollen tubes appeared in both the “Top” and “Middle” styles. A clear distinction exists among the pollen tubes growing from the incision of the three styles’ lengths, with the shortest having the most pollen tubes and growing longer. However, no pollen tubes grew out of the incision in the “bottom” style, even after extending to 96 HAP. Moreover, the presence of the ovary seemed to have no noticeable effect on the pollen tube growth (**Figure 5B**).

The above device was incompletely simulated, as the YZ2 pollen tube grew *in situ* at the base of the HS’s style, the upper end of the ovary. Adding calcium to the medium became an approach, as the proper amount catalyzes pollen tube growth. The final determination was achieved by comparing different concentration gradients and adding 50 mg·L^-1^ CaCl_2_ (**Supplementary Table 1**) to the medium to ensure that the pollen tube of YZ2 grows out of the incision of the “bottom” style. The entire procedure of the semi-*in vivo* experiment is illustrated in **Figure 5C**.

#### 3.4.2 Calcium ion gradients at the tips of pollen tubes *in vitro* or semi-*in vivo*

To observe the growth of interspecific incompatible combination “HS × YZ2” *in vivo*, Fluo-4/AM was loaded into pollen tubes with compatible combination “HS × HX” and HX and YZ2 pollen tubes cultured *in vitro* as controls.

**Figure 6:**
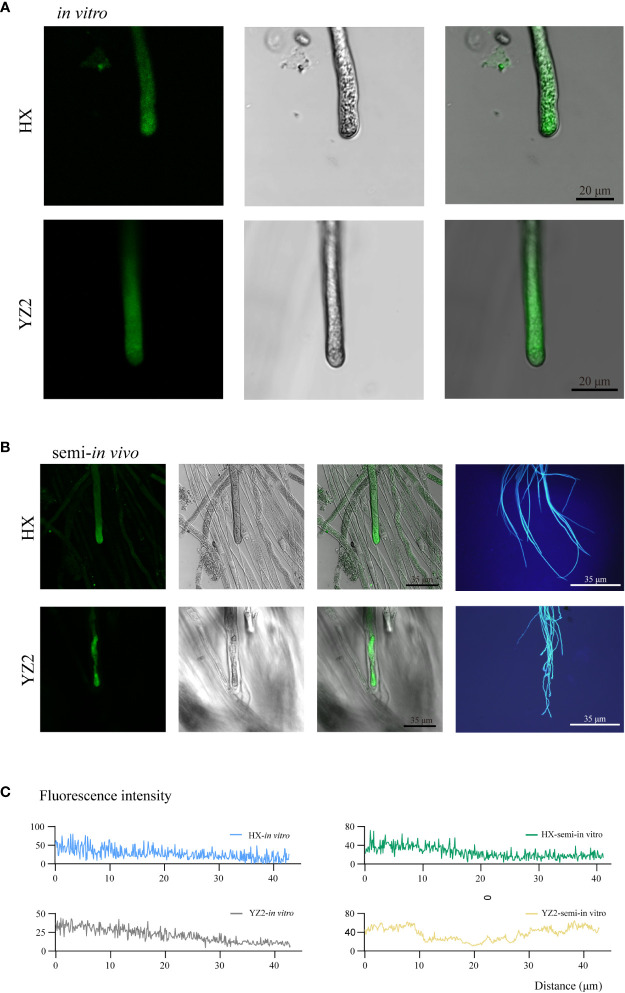
Characterization of calcium ion distribution at the tip of pollen tubes *in vitro* or semi-*in vivo*. **(A)** Calcium ion gradient distribution at the tip of HX and YZ2 pollen tubes *in vitro*. From left to right were the fluorescent field, bright field, and merged image. **(B)** Distribution of calcium ion gradients at the tips of HX and YZ2 pollen tubes and pollen tube morphology in semi-*in vivo* system. From left to right, the fluorescence field, bright field, merged image, and aniline blue fluorescence image. **(C)** Fluorescence intensity of HX and YZ2 pollen tube tips *in vitro* and in semi-*in vivo*. The measurement distance was about 40 μm from the tip of the pollen tube.

A gradient-changing [Ca^2+^]_cyt_ fluorescence was seen in the cytoplasm of the *in vitro* cultured HX pollen tubes after the subapical region, and YZ2 pollen tubes *in vitro* were similar (**Figure 6A**). However, the tips of YZ2 pollen tubes passing through the HS style developed an abnormal distribution of [Ca^2+^]_cyt_. Likewise, semi-*in vivo* cultured YZ2 pollen tubes exhibited a curled appearance that distinguished them from the smooth HX (**Figure 6B**). The fluorescence intensity of the pollen tube tips for the four treatments was recorded mentioned above, and the semi-*in vivo* cultured YZ2 pollen tube was distinguished from the other three pollen tubes (**Figure 6C**). The [Ca^2+^]_cyt_ gradient disappeared at the tips of YZ2 pollen tubes grown through HS styles, suggesting a strong inhibition effect of styles on pollen tubes.

## 4 Discussion

Pre-pollination barriers under natural conditions may be due to time, space, and availability of pollinators (Moyle et al., 2014). One of the main differences between the *C. oleifera* and *C. yuhsienensis* is flowering phenology, which hinders genetic exchange, despite the similarity of their pollinators and geographical location (Maekawa et al., 2022; Yuan et al., 2022). Artificial pollination overcame the flowering asynchronism between *C. oleifera* and *C. yuhsienensis* and facilitated the genetic exchange between them. *C. yuhsienensis* is highly resistant to anthracnose and possesses many excellent traits, such as flowering in spring, the higher unsaturated fatty acid content of its seeds, and fragrance of the flowers (Nie et al., 2020), which offer us the possibility of improving flowering time, oil quality, and ornamental properties. Gene exchange between *C. oleifera* and *C. yuhsienensis* can be facilitated through artificial crosses, and the favorable traits of both can be effectively combined to further breed premium quality oil in genus *Camellia*. Combining abilities also differ among different genotypes with the same species in the present study. The hybrids of crosses with different genotypes in these two species will be expanded, to further elucidate the genetic mechanism of UCI, which is of great significance in *C. oleifera* tree production and breeding.

After pollination, the pre-zygotic barrier is mainly characterized by pollen tubes. The rate of pollen germination in different pistils, as well as pollen tube growth, varies widely. In the style of *Nicotiana longiflora*, the pollen tube growth rate of interspecific hybrids was higher than that of self-crosses, while in *N. plumbaginifolia*, the growth rates of the two pollen tubes were not significantly different (Figueroa-Castro and Holtsford, 2009). In *Petunia*, pollen tubes for intraspecific pollination grow more rapidly than those for interspecific growth (Kato et al., 2022). In interspecific hybrids of the tomato clade, pollen tubes of distinct species have different growth rate in the pistil of the same species (Baek et al., 2015). Self-incompatibility is prevalent in the genus *Camellia*, and the pollen tube of cross-pollination grew faster in the style than self-pollination (Liao et al., 2014). Both cross- and self-pollinated pollen tubes reached the base of the style at 48 HAP (Liao et al., 2014), whereas in our work in interspecific crossed with *C. yuhsienensis* pollen tubes reach only 30% of the style. The growth rate of pollen tubes is related to the mating system of the species, with interspecific pollen tubes will be suppressed at a high level in the pistil (Tonnabel et al., 2021). The HS pollen tubes growing in the style of *C. yuhsienensis* were bright and numerous under the fluorescence microscope, while those growing in the style of *C. oleifera* were relatively small and dark, which correlated with the characteristics of the pollen itself (Shivanna, 1982). In this study, the incompatible pollen tubes of interspecific crosses of the genus *Camellia* exhibited morphologies distinct from the compatible pollen tubes, such as swelling, branching, and twisting (Nikolic and Milatovic, 2010), which were similar to the abnormal pollen tubes of its self-pollination (Liao et al., 2014). UCI and SI are potentially linked in some flowering plants (Lewis and Crowe, 1958; Hancock et al., 2003), but it is unknown in genus *Camellia*. Many species have yielded such findings on the mechanism of hybridization disorder recently of UCI and SI (He et al., 2019; Takada et al., 2021; Wang et al., 2022), but the molecular mechanism of cross-incompatibility in genus *Camellia* still requires further elucidation to provide insights for overcoming the reproductive barriers among genus *Camellia* species, and to propose reliable methods to overcome as well.

Post-zygotic barriers usually lead to fertilization or stagnation at various stages of development (Chen et al., 2016). Even though double fertilization and globular embryos have been observed in the embryo sacs of interspecies-pollinated *Chimonanthus*, the embryos were eventually aborted due to undeveloped endosperm (Wang et al., 2014). The structure of unfertilized ovules and the double fertilization process we observed are consistent with the previous research (Gao et al., 2015). The fertilized ovules of the hybrid combination “*C. oleifera* × *C. yuhsienensis*” were successfully developed, albeit rather sparsely, suggesting that the failure of the pollen tube entering the embryo sac is a key point leading to its incompatibility. Unfertilized ovules were distinguished from fertilized ovules by turning brown and aborted (Liao et al., 2014; Hu et al., 2020). In the above cross combinations, the volume difference between the sparsely fertilized ovules and the unfertilized ovules became progressively larger during maturation, giving rise to uneven fruit enlargement. Even though “*C. oleifera* × *C. yuhsienensis*” overcame a strong pre-zygotic barrier to obtain fruit, only one fertile seed was likely available (**Supplementary Figure 2**).

Semi-*in vivo* pollination assays provide an effective method for studying pollen tubes *in vivo*. In exploring the conditions of this experiment, we found that style length had a remarkable effect on pollen tube growth, as evidenced by the extraordinary ability of interspecific pollen tubes to grow after the top-style. Due to the short length of YZ2’s style, physical isolation may be a factor (Lee et al., 2008). No pollen tubes could be visualized at the incision of the intact style before modified medium at any time after 24 HAP. Dissecting the styles for staining revealed that many pollen tubes had grown to the base at 36 HAP, but failed to grow outside the incision. After adding exogenous calcium, the pollen tubes were allowed to grow out of the incision, implying that calcium alleviated the inhibition of HS styles to the interspecific pollen tubes. Pollen tube growth is regulated by dynamic calcium ion concentration, promoted by certain calcium concentrations (Steinhorst and Kudla, 2013). The reason for inhibition of incompatible pollen tube growth is hypothesized to be a large influx of extracellular calcium suppressing growth, resulting in abnormal pollen tube tip morphology (Guan et al., 2013). Consequently, the ion changes were measured before and after pollination of pistils from oil camellia cross combinations, similar to the tea tree pollination studies (Ma et al., 2018). The calcium content of the pistil showed an irregular change after pollination compared to compatible pollination (**Supplementary Figure 3**). However, no further evidence is available to suggest that the variation in calcium content is the main cause of UCI and additional research is still requested.

The ovary factor in the semi-*in vivo* experiment exploration was set up to clarify whether the ovary affects the pollen tube growing to the base in pollen-pistil interactions, like affecting self-incompatible pollen tubes (Chang et al., 2023). Although the inhibition of the pollen tube by the style was more pronounced in this assay, the ovary should not be considered without an effect, which needs to be determined by further experiments. In conclusion, the semi-*in vivo* pollination test plays a role in pollination compatibility study. It allows trials on more species and genotypic materials. This research provides new guidance and reference for overcoming the barriers to hybridization in oil tea and the mechanism of interspecific hybrid incompatibility.

## 5 Conclusion

UCI was prevalent between *C. oleifera* and *C. yuhsienensis*, and artificial pollination overcame the spatial and temporal barriers. The growth of pollen tubes *in vivo* revealed that UCI in *C. oleifera* and *C. yuhsienensis* was a pre-zygotic barrier, manifested by the slowed growth rate of pollen tubes in *C. yuhsienensis* when *C. oleifera* was the recipient parent, and the swelling appeared at the base of the style. Semi-*in vivo* system clarified further that the style inhibited interspecific pollen tube growth and disrupt the intracellular calcium gradient in the pollen tube. To our knowledge, this is the first time that the cytological mechanism of UCI is reported in *C. oleifera* and *C. yuhsienensis.*

## Data availability statement

The original contributions presented in the study are included in the article/**Supplementary Material**. Further inquiries can be directed to the corresponding author.

## Author contributions

HG and YC contributed to conception and design of the study. HG, JX and YC did the experiments and collected the samples. HG and XY performed the statistical analysis. HG wrote the original draft of the manuscript. WG and YC edited the manuscript. All authors contributed to the article and approved the submitted version.

## References

[B1] Baack E. Melo M. C. Rieseberg L. H. Ortiz-Barrientos D. (2015). The origins of reproductive isolation in plants. New Phytol. 207, 968–984. doi: 10.1111/nph.13424 25944305

[B2] Baek Y. S. Covey P. A. Petersen J. J. Chetelat R. T. McClure B. Bedinger P. A. (2015). Testing the SI × SC rule: pollen–pistil interactions in interspecific crosses between members of the tomato clade ( *Solanum* section *Lycopersicon* , solanaceae). Am. J. Bot. 102, 302–311. doi: 10.3732/ajb.1400484 25667082

[B3] Bittencourt N. S. (2019). Reproductive systems and low outbreeding barriers between *Jacaranda cuspidifolia* and *J. mimosifolia* (Jacarandeae, bignoniaceae). Nordic J. Bot. 37, njb.02558. doi: 10.1111/njb.02558

[B4] Chang Y. Gong W. Xu J. Gong H. Song Q. Xiao S.. (2023). Integration of semi- *in vivo* assays and multi-omics data reveals the effect of galloylated catechins on self-pollen tube inhibition in *Camellia oleifera* . Horticulture Res. 10, uhac248. doi: 10.1093/hr/uhac248 PMC9832949 36643738

[B5] Chen X. Jiang L. Bao A. Liu C. Liu J. Zhou G. (2021). Molecular characterization, pathogenicity and biological characterization of colletotrichum species associated with anthracnose of camellia yuhsienensis hu in China. Forests 12, 1712. doi: 10.3390/f12121712

[B6] Chen C. Zhiguo E. Lin H.-X. (2016). Evolution and molecular control of hybrid incompatibility in plants. Front. Plant Sci. 7. doi: 10.3389/fpls.2016.01208 PMC4980391 27563306

[B7] Claessen H. Van de Poel B. Keulemans W. De Storme N. (2022). A semi *in vivo* pollination technique to assess the level of gametophytic self-incompatibility and pollen tube growth in pear (*Pyrus communis* l.). Plant Reprod. 35, 127–140. doi: 10.1007/s00497-021-00435-y 35032190

[B8] Figueroa-Castro D. M. Holtsford T. P. (2009). Post-pollination mechanisms in *Nicotiana longiflora* and n. plumbaginifolia: pollen tube growth rate, offspring paternity and hybridization. Sex Plant Reprod. 22, 187–196. doi: 10.1007/s00497-009-0103-6 20033439

[B9] Gao C. Yang R. Yuan D. (2018). Structural characteristics of the mature embryo sac of *Camellia oleifera* . Nordic J. Bot. 36, njb–01673. doi: 10.1111/njb.01673

[B10] Gao C. Yuan D. Yang Y. Wang B. Liu D. Zou F. (2015). Pollen tube growth and double fertilization in *Camellia oleifera* . J. Amer. Soc Hortic. Sci. 140, 12–18. doi: 10.21273/JASHS.140.1.12

[B11] Goulet B. E. Roda F. Hopkins R. (2017). Hybridization in plants: old ideas, new techniques. Plant Physiol. 173, 65–78. doi: 10.1104/pp.16.01340 27895205 PMC5210733

[B12] Guan Y. Guo J. Li H. Yang Z. (2013). Signaling in pollen tube growth: crosstalk, feedback, and missing links. Mol. Plant 6, 1053–1064. doi: 10.1093/mp/sst070 23873928 PMC3842152

[B13] Hafidh S. Potěšil D. Fíla J. Feciková J. Čapková V. Zdráhal Z.. (2014). In search of ligands and receptors of the pollen tube: the missing link in pollen tube perception. Biochem. Soc. Trans. 42, 388–394. doi: 10.1042/BST20130204 24646249

[B14] Hancock C. N. Kondo K. Beecher B. McClure B. (2003). The *S* –locus and unilateral incompatibility. Phil. Trans. R. Soc Lond. B 358, 1133–1140. doi: 10.1098/rstb.2003.1284 12831479 PMC1693195

[B15] He D. Lou X.-Y. He S.-L. Lei Y.-K. Lv B.-V. Wang Z.. (2019). Isobaric tags for relative and absolute quantitation-based quantitative proteomics analysis provides novel insights into the mechanism of cross-incompatibility between tree peony and herbaceous peony. Funct. Plant Biol. 46, 417. doi: 10.1071/FP18163 30940329

[B16] Hu G. Gao C. Fan X. Gong W. Yuan D. (2020). Pollination compatibility and Xenia in *Camellia oleifera* . horts 55, 898–905. doi: 10.21273/HORTSCI14933-20

[B17] Kato M. Watanabe H. Hoshino Y. (2022). Evaluation of pollen tube growth ability in *Petunia* species having different style lengths. Plant Biotechnol. 39, 85–92. doi: 10.5511/plantbiotechnology.21.1113a PMC9300434 35937536

[B18] Lee C. B. Page L. E. McClure B. A. Holtsford T. P. (2008). Post-pollination hybridization barriers in *Nicotiana* section alatae. Sex Plant Reprod. 21, 183–195. doi: 10.1007/s00497-008-0077-9

[B19] Lenzi A. Biricolti S. Vivoli R. Bulleri F. Baldi A. (2021). Cross-breeding program in the genus *Camellia* involving the “everblooming” camellia ( c. azalea C.F. wei). Acta Hortic. 1331, 35–42. doi: 10.17660/ActaHortic.2021.1331.4

[B20] Lewis D. Crowe L. K. (1958). Unilateral interspecific incompatibility in flowering plants. Heredity 12, 233–256. doi: 10.1038/hdy.1958.26

[B21] Li K. Wang Y. Qu H.. (2018). Effect of vacuum infiltration treatment on the loading of fluorescent dye into pollen tubes. Acta Botanica Boreali-Occidentalia Sin. 38, 2138–2147. doi: 10.7606/j.issn.1000-4025.2018.11.2138

[B22] Li J. Zhang C. Qu X. Luo Z. Lu S. Kuzyakov Y.. (2021). Microbial communities and functions in the rhizosphere of disease-resistant and susceptible camellia spp. Front. Microbiol. 12. doi: 10.3389/fmicb.2021.732905 PMC8558623 34733251

[B23] Liao T. Yuan D.-Y. Zou F. Gao C. Yang Y. Zhang L.. (2014). Self-sterility in *Camellia oleifera* may be due to the prezygotic late-acting self-incompatibility. PLoS One 9, e99639. doi: 10.1371/journal.pone.0099639 24926879 PMC4057179

[B24] Lowry D. B. Modliszewski J. L. Wright K. M. Wu C. A. Willis J. H. (2008). The strength and genetic basis of reproductive isolating barriers in flowering plants. Phil. Trans. R. Soc B 363, 3009–3021. doi: 10.1098/rstb.2008.0064 18579478 PMC2607309

[B25] Ma Q. Chen C. Zeng Z. Zou Z. Li H. Zhou Q.. (2018). Transcriptomic analysis between self- and cross-pollinated pistils of tea plants (*Camellia sinensis*). BMC Genomics 19, 289. doi: 10.1186/s12864-018-4674-1 29695246 PMC5918555

[B26] Maekawa R. Mitani T. Ishizaki S. Kubota S. Ohara M. (2022). Asymmetrical hybridization between trillium apetalon and t. tschonoskii for the formation of a hybrid *T. miyabeanum* (Melanthiaceae). Plant Syst. Evol. 308, 13. doi: 10.1007/s00606-022-01806-2

[B27] Maune J. F. Camadro E. L. Erazzú L. E. (2018). Cross-incompatibility and self-incompatibility: unrelated phenomena in wild and cultivated potatoes? Botany 96, 33–45. doi: 10.1139/cjb-2017-0070

[B28] Miller D. D. Callaham D. A. Gross D. J. Hepler P. K. (1992). Free Ca^2+^ gradient in growing pollen tubes of lillium. J. Cell Sci. 101, 7–12. doi: 10.1242/jcs.101.1.7

[B29] Moyle L. C. Jewell C. P. Kostyun J. L. (2014). Fertile approaches to dissecting mechanisms of premating and postmating prezygotic reproductive isolation. Curr. Opin. Plant Biol. 18, 16–23. doi: 10.1016/j.pbi.2013.12.005 24457825

[B30] Muñoz-Sanz J. V. Zuriaga E. Cruz-García F. McClure B. Romero C. (2020). Self-(In)compatibility systems: target traits for crop-production, plant breeding, and biotechnology. Front. Plant Sci. 11. doi: 10.3389/fpls.2020.00195 PMC7098457 32265945

[B31] Nie Z. Huang X. Hu Z. Li X. Yin H. Li J. (2020). Characterization of the complete chloroplast genome of *Camellia yuhsienensis* hu, a resilient shrub with strong floral fragrance. Mitochondrial DNA Part B 5, 2998–2999. doi: 10.1080/23802359.2020.1797580 33458032 PMC7783147

[B32] Nikolic D. Milatovic D. (2010). Examining self-compatibility in plum (*Prunus domestica* l.) by fluorescence microscopy. Genetika 42, 387–396. doi: 10.2298/GENSR1002387N

[B33] Okamoto A. Ureshino K. (2015). Pre- and post-fertilization barriers in interspecific hybridization between evergreen azalea species and *Rhododendron uwaense* h. hara & t. yamanaka. Hortic. J. 84, 355–364. doi: 10.2503/hortj.MI-036

[B34] Quan W. Wang A. Gao C. Li C. (2022). Applications of Chinese *Camellia oleifera* and its by-products: a review. Front. Chem. 10. doi: 10.3389/fchem.2022.921246 PMC9171030 35685348

[B35] Shivanna K. R. (1982). “Pollen-pistil interaction and control of fertilization,” in Experimental embryology of vascular plants. Ed. Johri B. M. (Berlin, Heidelberg: Springer Berlin Heidelberg), 131–174. doi: 10.1007/978-3-642-67798-4_7

[B36] Steinhorst L. Kudla J. (2013). Calcium - a central regulator of pollen germination and tube growth. Biochim. Biophys. Acta (BBA) - Mol. Cell Res. 1833, 1573–1581. doi: 10.1016/j.bbamcr.2012.10.009 23072967

[B37] Takada Y. Mihara A. He Y. Xie H. Ozaki Y. Nishida H.. (2021). Genetic diversity of genes controlling unilateral incompatibility in Japanese cultivars of Chinese cabbage. Plants 10, 2467. doi: 10.3390/plants10112467 34834830 PMC8619800

[B38] Tonnabel J. David P. Janicke T. Lehner A. Mollet J.-C. Pannell J. R.. (2021). The scope for postmating sexual selection in plants. Trends Ecol. Evol. 36, 556–567. doi: 10.1016/j.tree.2021.02.013 33775429

[B39] Wang J. Chen J. Huang S. Han D. Li J. Guo D. (2022). Investigating the mechanism of unilateral cross incompatibility in longan (*Dimocarpus longan* lour.) cultivars (Yiduo × shixia). Front. Plant Sci. 12. doi: 10.3389/fpls.2021.821147 PMC8874016 35222456

[B40] Wang Y. Chen J. Li D.-W. Zheng L. Huang J. (2017). CglCUT1 gene required for cutinase activity and pathogenicity of colletotrichum gloeosporioides causing anthracnose of *Camellia oleifera* . Eur. J. Plant Pathol. 147, 103–114. doi: 10.1007/s10658-016-0983-x

[B41] Wang W. Zhou L. Huang Y. Bao Z. Zhao H. (2014). Reproductive barriers in interspecific hybridizations among *Chimonanthus praecox* (L.) link, c. salicifolius s. y. hu, and *C. nitens* Oliver from pollen–pistil interaction and hybrid embryo development. Scientia Hortic. 177, 85–91. doi: 10.1016/j.scienta.2014.07.040

[B42] Wei H. Gao C. Qiu J. Long L. Wang B. Yang L.. (2021). Flowering biological characteristics of *Camellia weiningensis* Y.K. Li. horts 56, 1331–1339. doi: 10.21273/HORTSCI16024-21

[B43] Xu H. Li F. Pan Y. Gong X. (2017). Interspecific hybridization processes between *Michelia yunnanensis* and m. crassipes and embryogenesis of the heterozygote. horts 52, 1043–1047. doi: 10.21273/HORTSCI12086-17

[B44] Yuan B. Hu G.-X. Zhang X.-X. Yuan J.-K. Fan X.-M. Yuan D.-Y. (2022). What are the best pollinator candidates for *Camelia oleifera*: do not forget hoverflies and flies. Insects 13, 539. doi: 10.3390/insects13060539 35735876 PMC9224817

[B45] Zheng R. Su S. Xiao H. Tian H. (2019). Calcium: a critical factor in pollen germination and tube elongation. IJMS 20, 420. doi: 10.3390/ijms20020420 30669423 PMC6358865

