# Supplementary figures and images for: Unilateral cross-incompatibility between Camellia oleifera and C. yuhsienensis provides new insights for hybridization in Camellia spp
Source: Front Plant Sci. 2023 Jul 3;14:1182745. doi: 10.3389/fpls.2023.1182745 (PMC10350491; doi:10.3389/fpls.2023.1182745)

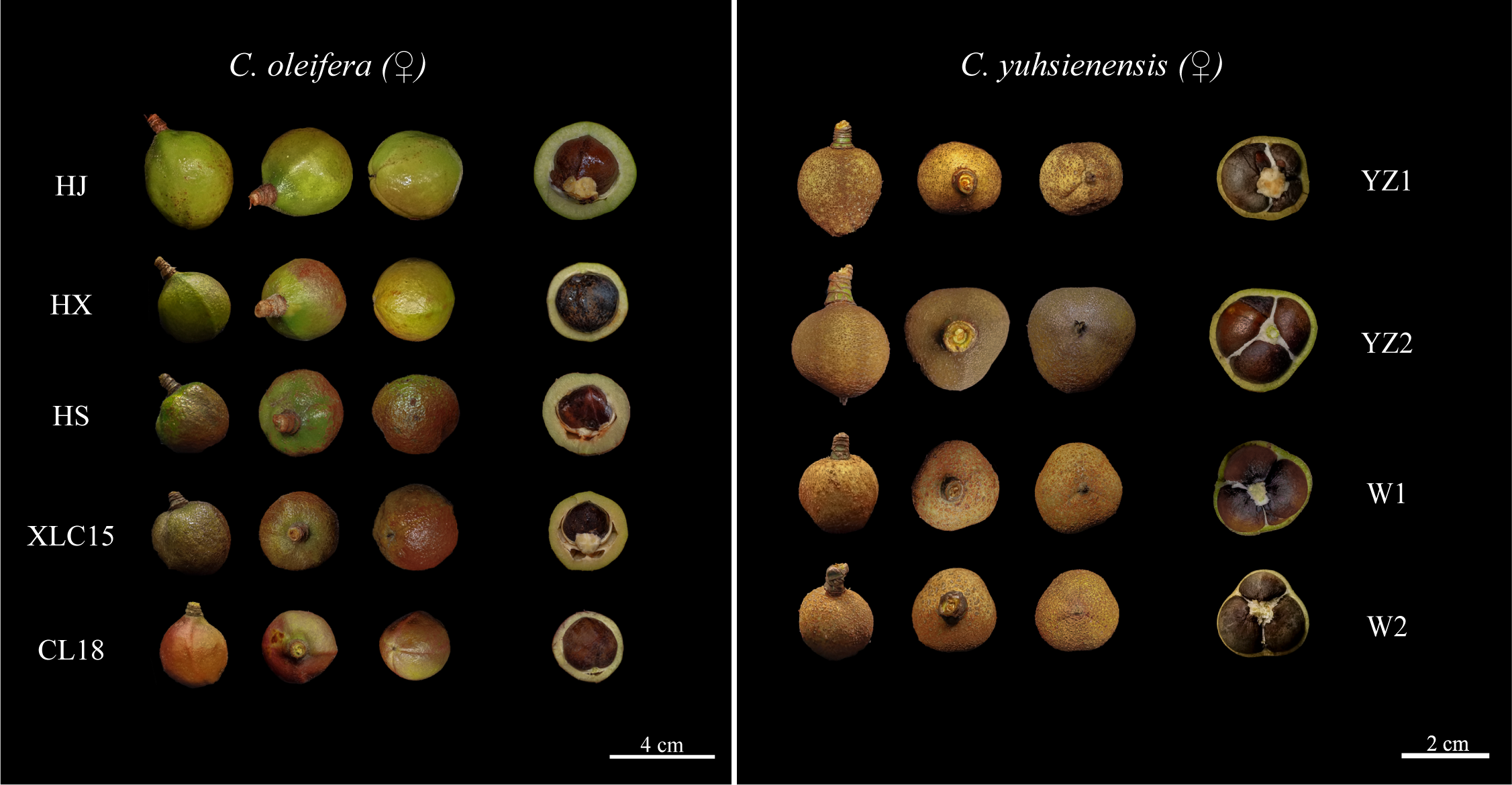

Supplement: Supplementary file 1 [file Image_1.tif]

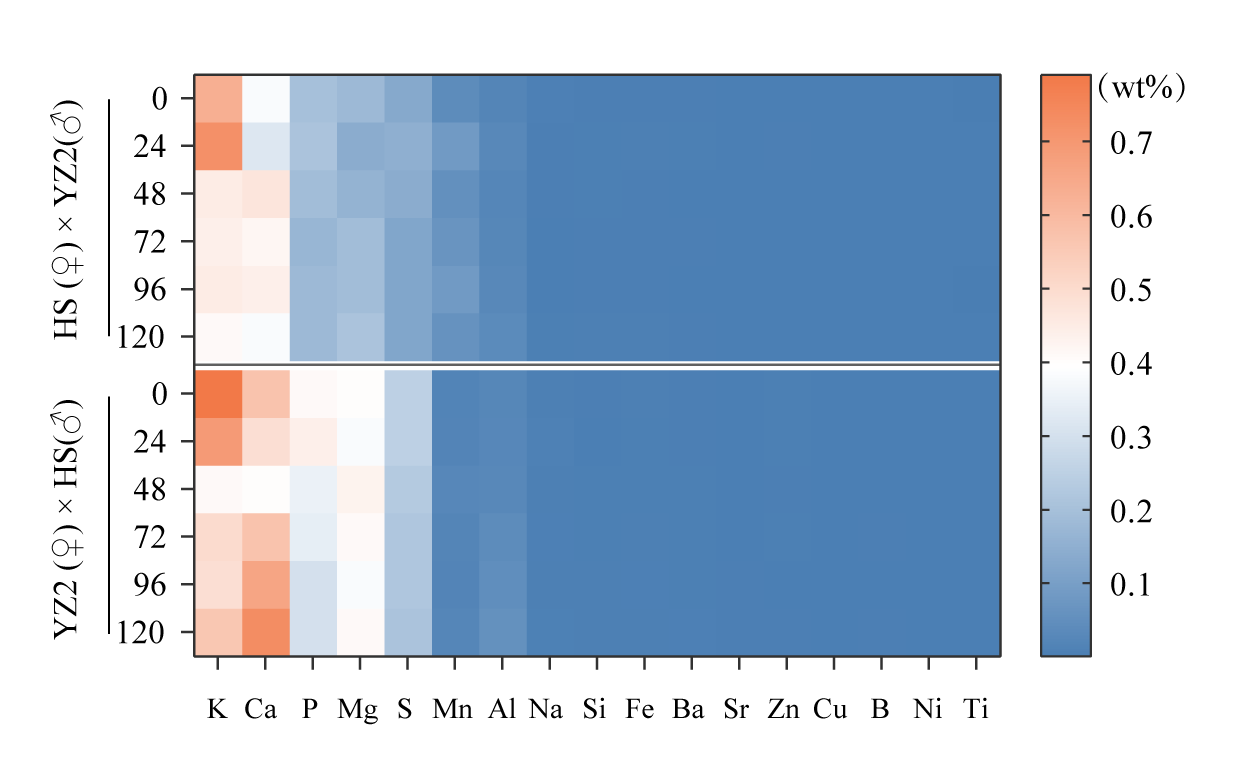

Supplement: Supplementary file 2 [file Image_2.tif]
